# Clinicopathological Correlation of Immunohistochemical Markers, Serum Biomarkers, and Masticatory Muscle Activity With the Severity of Oral Submucous Fibrosis

**DOI:** 10.7759/cureus.99679

**Published:** 2025-12-19

**Authors:** Ajeena M M, Vikas Gupta, Utkal P Mishra, Ganakalyan Behera, Ruchi Singh, Garima Goel, Aman Kumar, Anjan K Sahoo, Shaila Sidam

**Affiliations:** 1 Burns and Plastic Surgery, All India Institute of Medical Sciences, Patna, Patna, IND; 2 Otolaryngology-Head and Neck Surgery, All India Institute of Medical Sciences, Bhopal, Bhopal, IND; 3 Physiology, All India Institute of Medical Sciences, Bhopal, Bhopal, IND; 4 Pathology, All India Institute of Medical Sciences, Bhopal, Bhopal, IND; 5 Interventional Radiology, All India Institute of Medical Sciences, Bhopal, Bhopal, IND

**Keywords:** e-cadherin, electromyography, immunohistochemical marker, masticatory muscle, oral squamous cell carcinoma, serum iron, sox-2, submucous fibrosis, ultrasonography

## Abstract

Background: Oral submucous fibrosis (OSMF) is a chronic, progressive condition recognised for its potential to undergo malignant transformation. Molecular biomarkers and functional muscle alterations may help predict disease progression and risk of transformation to oral squamous cell carcinoma (OSCC).

Objective: To evaluate immunohistochemical (IHC) expression of p63, Ki-67, SOX2, and E-cadherin; correlate their expression with clinical and histological grades of OSMF; assess serum iron and protein levels; and analyse masticatory muscle thickness and electromyographic activity.

Methods: A cross-sectional study was conducted over 18 months, including 30 OSMF patients, five healthy controls, and five OSCC patients. Clinical grading (Mehrotra), histological grading (Khanna and Andrade), immunohistochemical expression of p63, Ki-67, SOX2, and E-cadherin markers, serum biochemical profiling, ultrasonographic measurement of muscle thickness, and needle electromyography were performed.

Results: E-cadherin expression showed a significant and gradual reduction from normal mucosa to OSMF and further to OSCC (p = 0.017), whereas p63, Ki-67, and SOX2 exhibited significant progressive increases across the same continuum (p < 0.001). However, none of these markers showed a statistically significant correlation with the clinical or histological grades of OSMF. Serum iron, ferritin, and total protein parameters did not exhibit significant associations with the clinical grade of OSMF. In contrast, ultrasonography revealed a significant decline in the thickness of the masseter, temporalis, and orbicularis oris muscles with advancing clinical grades of OSMF (p < 0.05), indicating progressive myofascial involvement. Electromyographic findings corroborated these observations, with a reduction in motor unit action potential duration in the masseter muscle across advanced grades, reflecting underlying myopathic changes associated with fibrosis.

Conclusion: p63, Ki-67, SOX2, and E-cadherin demonstrate significant expression across normal mucosa, OSMF, and OSCC, supporting their role as markers of malignant transformation. However, these biomarkers do not reliably reflect disease severity within OSMF. Ultrasonographic and electromyography (EMG) assessments correlate with clinical progression and may serve as valuable adjuncts for early detection of muscle dysfunction in OSMF.

## Introduction

Oral submucous fibrosis (OSMF) is a chronic, insidious, potentially malignant disorder characterised by juxta-epithelial inflammation and progressive fibrosis of the oral cavity, often extending to the pharynx and upper oesophagus [1]. OSMF represents a significant public health concern in South and Southeast Asia, particularly India, where widespread availability and consumption of areca nut, betel quid, gutka, and other smokeless tobacco products have driven a rapid rise in disease prevalence. Its malignant transformation rate ranges from 7% to 30%, and is higher than that of leukoplakia [2]. The malignant transformation potential of OSMF varies depending on the patient's risk factors, including the duration of exposure to tobacco or betel quid, as well as clinical and histological features. Several molecular pathways, including the aberrant expression of p53-related proteins, proliferation markers, and adhesion molecules, contribute to disease progression and the risk of transformation to oral squamous cell carcinoma (OSCC) [3].

Although extensive molecular data exist for leukoplakia, fewer studies have explored biomarker expression in OSMF [4,5]. Assessment of proliferation markers (Ki-67), epithelial stemness factors (SOX2), cell adhesion molecules (E-cadherin), and basal cell markers (p63) may aid in stratifying the risk of malignant transformation. Sex-determining region Y-box 2 (SOX2) is an embryonic transcription factor that plays a central role in maintaining stem cell pluripotency and supporting the structural and functional integrity of squamous epithelium. Its aberrant regulation has gained increasing attention in the context of oral carcinogenesis [6]. In oral squamous cell carcinoma (OSCC), higher SOX2 expression is frequently observed in well-differentiated tumours, suggesting that SOX2 may be associated with a more differentiated epithelial phenotype and correspondingly lower rates of lymph node metastasis, as well as better clinical outcomes [7]. Conversely, reduced or absent SOX2 expression has been proposed as an independent marker of poor prognosis, reflecting a shift toward a less differentiated and more aggressive tumour biology.

Additionally, chronic areca nut chewing alters masticatory muscle dynamics. The current literature provides conflicting evidence on whether restricted mouth opening is primarily due to mucosal fibrosis or also due to changes in muscle thickness and function [8]. Ultrasonography and electromyography (EMG) provide objective means to evaluate muscle morphology and activity and may provide insights into the functional consequences of OSMF, as supported by Saker et al., who demonstrated differences in masticatory muscle activity across skeletal malocclusion types [9].

Nutritional deficiencies, particularly reduced serum iron levels, have also been implicated in the pathogenesis of OSMF [10]. Reduced iron availability compromises epithelial integrity and collagen metabolism. Alterations in serum protein levels have also been reported, although their correlation with disease severity is inconsistent [11].

In this context, the present study aimed to comprehensively evaluate immunohistochemical expression of p63, Ki-67, SOX2, and E-cadherin; assess serum iron and total protein levels; and analyse ultrasonographic and electromyographic parameters of key masticatory muscles, correlating all findings with the clinical and histopathological grades of OSMF. We hypothesised that aberrant expression of these molecular markers, nutritional deficiencies, and muscle dysfunction is associated with more advanced clinical and histopathological grades of OSMF. Normal oral mucosa and OSCC specimens were included as controls to serve both as diagnostic anchors and statistical comparators for interpreting marker expression patterns.

## Materials and methods

This cross-sectional observational study was conducted over 18 months between 1st March 2021 and 31st August 2022 in the Department of Otorhinolaryngology and Head & Neck Surgery at a tertiary care referral centre in central India. The study commenced after obtaining approval from the Institutional Human Ethics Committee. The study population comprised patients attending the ENT outpatient department who were clinically diagnosed with oral submucous fibrosis (OSMF). Written informed consent was obtained from all participants. In this study, we did not propose to test any formal hypothesis. This was an exploratory study that would serve as a hypothesis study if we found a correlation between clinical and pathological grade. Therefore, a logistically feasible sample was taken. Based on the inclusion criteria, 30 clinically diagnosed OSMF patients were enrolled in the study group for continuous measurement. For comparative analysis, two additional groups were included: five normal control subjects who required minor oral biopsies for benign lesions, such as fibromas or mucoceles, and five positive controls with biopsy-proven oral squamous cell carcinoma (OSCC). Patients previously treated for OSMF or OSCC, those with systemic diseases affecting muscle or immune function, and individuals unwilling to participate were excluded from the study. A detailed overview of the comprehensive inclusion and exclusion criteria is presented in Table 1.

**Table 1:** Inclusion and exclusion criteria.

Inclusion criteria	Exclusion criteria
Patients clinically diagnosed with oral submucous fibrosis (OSMF)	Patients with previous treatment for OSMF (medical or surgical).
Age 18 years and above.	Patients with coexisting oral lesions such as leukoplakia, erythroplakia, or lichen planus.
Patients willing to undergo biopsy, ultrasonography, electromyography, and blood investigations.	Individuals with pre-existing systemic conditions affecting muscle physiology (e.g., neuromuscular disorders, metabolic myopathies).
Patients providing written informed consent.	Pregnant or lactating women.

**Figure 1:**
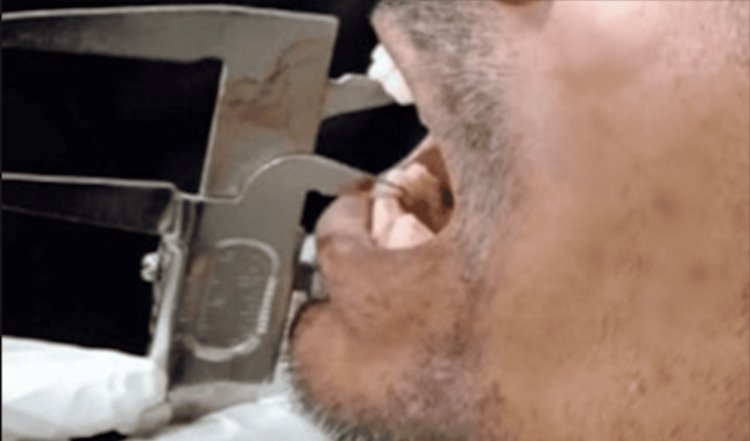
Measurement of inter-incisoral distance using Vernier calipers.

A detailed history was obtained for each participant, including demographic information, duration and frequency of areca nut or tobacco use, relevant systemic illnesses, and presenting symptoms such as burning sensation, restricted mouth opening, or trismus. Clinical examination included inspection and palpation of the oral mucosa to assess blanching, fibrous bands, ulceration, and involvement of the buccal mucosa, soft palate, faucial pillars, and floor of the mouth. Inter-incisoral mouth opening was measured at the point of maximum painless opening using Vernier calipers, and patients were categorised into four clinical stages based on Mehrotra et al.'s classification (Figure 1).

According to Mehrotra et al., oral submucous fibrosis is clinically classified into four grades based on the severity of symptoms, the extent of mucosal involvement, and the degree of mouth opening restriction. Grade I represents the initial stage, characterised by stomatitis, burning sensation in the buccal mucosa, and absence of palpable fibrous bands. Grade II shows progression, characterised by a persistent burning sensation, the formation of fibrous bands, involvement of the soft palate, and a reduction in mouth opening to 26-35 mm. Grade III includes all features of Grade II, along with blanching of the oral mucosa, involvement of the tongue, and a further decrease in mouth opening to 6-25 mm. Grade IV is the most advanced stage, presenting with fibrosis of the lips, extensive mucosal involvement, and severely restricted mouth opening of approximately 5 mm.

Incisional biopsies were obtained from representative fibrotic areas of clinically diagnosed OSMF cases under aseptic conditions and local anaesthesia. Tissues were fixed in 10% buffered formalin, processed, and stained with haematoxylin and eosin. Histopathological grading was performed according to the Khanna and Andrade classification, which categorises OSMF into four grades based on epithelial thickness, collagen deposition, hyalinisation, and inflammatory infiltrate [12]. All OSMF samples were further subjected to immunohistochemical (IHC) analysis for four molecular markers: p63, Ki-67, SOX2, and E-cadherin. The following primary antibodies were used for immunohistochemical analysis: Ki-67 (Clone MIB-1, Mouse monoclonal antibody, PM210, 6 mL RTU, PathnSitu, Livermore, California, USA), p63 (Clone 4A4, Mouse monoclonal antibody, PM105, 6 mL RTU, PathnSitu, Livermore, California, USA), SOX2 (Clone EP103, Rabbit monoclonal antibody, PR071, 6 mL RTU, PathnSitu, Livermore, California, USA), and E-cadherin (Clone EP6, Rabbit monoclonal antibody, PR039, 6 mL RTU, PathnSitu, Livermore, California, USA). All antibodies were ready-to-use formulations, and immunostaining was performed according to the manufacturer's standard protocols, including appropriate antigen retrieval, incubation, and detection steps. Four-micron sections were cut onto poly-L-lysine-coated slides, deparaffinized, rehydrated, and subjected to antigen retrieval using citrate buffer. Endogenous peroxidase was blocked, followed by incubation with primary antibodies. After washing, sections were treated with secondary antibodies and visualised using a diaminobenzidine (DAB) chromogen substrate. Two blinded pathologists independently assessed positivity, intensity, and distribution patterns. Scoring of immunohistochemical (IHC) markers was based on the percentage and intensity of positively stained epithelial cells. For p63 and SOX2, uniform brown nuclear staining was considered positive, while the absence of staining was regarded as negative. The expression was graded as 0 (negative), 1+ (1-25% positive cells), 2+ (26-50%), 3+ (51-75%), and 4+ (>75% positive cells). Ki-67 expression was similarly assessed, with brown-stained nuclei considered positive and scored using the same grading scale to calculate the labelling index. E-cadherin positivity was identified by membranous brown staining in dysplastic epithelial cells, while discontinuous or absent membrane staining (with or without cytoplasmic staining) was considered negative. Based on the percentage and intensity of membranous staining at 40× magnification, E-cadherin expression was scored as 0 (<10% positive cells), 1+ (10-25%, weak), 2+ (25-50%, mild to moderate), 3+ (50-75%, moderate to strong), and 4+ (>75%, very strong) [13].

Venous blood samples were collected from all participants after overnight fasting. Serum was separated and analysed using automated biochemical analysers for iron profile, including serum iron, ferritin, total iron-binding capacity (TIBC), and transferrin saturation, as well as total serum protein, albumin, globulin, and albumin-to-globulin ratio. These parameters were evaluated to determine any association between nutritional or haematologic status and disease severity.

**Figure 2:**
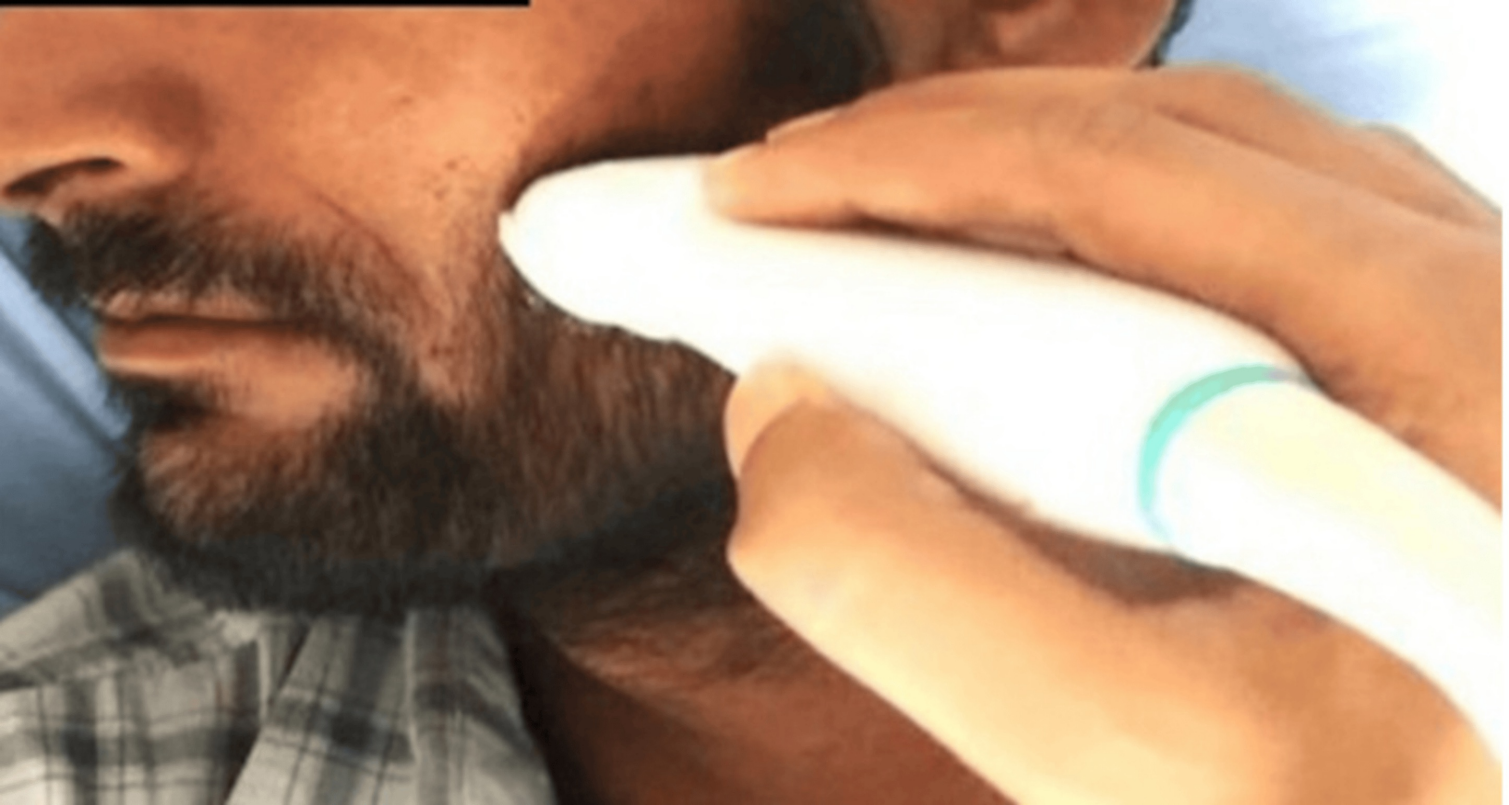
Ultrasonographic assessment of left masseter muscle thickness using a linear array transducer.

To assess the functional status of masticatory and perioral muscles, ultrasonography (USG) was performed using a high-resolution linear array transducer (Philips EPIQ 7, 5-12 MHz, Philips Healthcare, Bothell, WA, USA). Scanning was performed bilaterally with participants in a supine position, and the head was turned to provide optimal access to the probe. The transducer was positioned perpendicular to the ramus at the thickest portion of the masseter near the occlusal plane, over the anterior border of the hairline for the temporalis, and near the angle of the mouth for the orbicularis oris (Figure 2). Real-time imaging was obtained in a relaxed state, and muscle thickness was measured in millimetres using the electronic calliper tool within the ultrasound software. For each muscle, three standardised measurements were taken, and the mean value per side was recorded for analysis. All scans were performed by a single experienced radiologist who was blinded to the clinical and histopathological grades of the participants to minimise observer bias.

Electromyographic (EMG) recordings were performed using the Neuropack™electrophysiological apparatus in patients with OSMF and in five normal controls. Participants were seated upright with their heads in a neutral position, and their skin was cleansed with 70% alcohol. A 25 mm paediatric needle was used for needle EMG. The active and reference electrodes were placed at an interelectrode distance of 3-4 mm, and a ground electrode was placed on the forehead. Electrodes were positioned over the thickest region of the masseter (near the occlusal plane), the anterior temporalis (anterior to the hairline), and the orbicularis oris (near the mouth angle), confirmed by palpation during maximal voluntary contraction. Muscle activity was recorded in a relaxed state, with amplitude measured in microvolts and duration of motor unit action potentials in milliseconds. Filter settings were 20 Hz-10 kHz, gain 1 mV/div, and sweep speed 10 ms/div. Electrode placement and measurement protocols followed established methods described by Saker et al [9]. Amplitude (μV) and motor unit action potential duration (ms) were recorded systematically, compiled and tabulated for analysis.

Statistical analysis

All data were entered into Microsoft Excel (Microsoft Corporation, Redmond, Washington, USA) and analysed using the Statistical Package for the Social Sciences (SPSS) software, version 29.0 (IBM Corp., Armonk, NY, USA). Descriptive statistics, including mean, standard deviation, and percentage, were used to summarise the data. The Kruskal-Wallis rank sum test was applied to compare quantitative variables among the study groups, while Fisher’s exact test was used to assess associations between categorical variables. Missing data (<5%) were handled using pairwise deletion. A p-value of less than 0.05 was considered statistically significant. Correlations between clinical grade, histopathological grade, biochemical parameters, muscle thickness, EMG findings, and IHC marker expression were systematically explored. As this was an exploratory study, inferential statistics were applied to identify potential trends rather than to confirm hypotheses. No corrections for multiple testing were performed, and all p-values should be interpreted descriptively.

## Results

A total of 40 participants were included in the study, comprising 30 patients with oral submucous fibrosis (OSMF), five normal controls, and five biopsy-proven cases of oral squamous cell carcinoma (OSCC). Among the OSMF group, the majority were males, 32 (80%), while females constituted 8 (20%), reflecting the strong gender predisposition associated with areca nut consumption. Most patients belonged to the 35-50-year age group, 22 (70%), followed by those below 25 years of age, 8 (20%). The predominant presenting complaints among OSMF patients were a burning sensation in the oral cavity and progressive reduction in mouth opening. Tobacco was the most commonly used substance, reported by 22 (55%) patients, followed by areca nut (supari) in 6 (15%) and gutka in 3 (7.5%) patients. The mean age of starting substance abuse was around 22 years, which shows the gravity of the situation (Table 2).

**Table 2:** Demographic and clinical characteristics of OSMF patients. The data are presented as mean (standard deviation) and n (%). OSMF: oral submucous fibrosis.

Characteristics	Total (n = 40)	%
Gender		
Male	32	80.00
Female	8	20.00
Age	-	-
Below 25 years	8	20.00
25-35 years	6	15.00
35-50 years	22	70.00
Above 50 years	4	10.00
Duration of symptoms (in months)	25 (16.6)	-
Type of substance abuse		
Tobacco	22	55.0
Gutkha	3	7.50
Gutkha + tobacco both	1	2.50
Betel nut (supari)	6	15.00
Pan masala	1	2.50
Supari + gutkha	2	5
None	5	12.50
Duration of substance abuse	10.73 (4.51)	-
Years of substance abuse	9.73 (6.30)	-
Age of starting substance abuse	22.83 (6.84)	-
Clinical grade of OSMF		
Grade 1	5	16.67%
Grade 2	6	20%
Grade 3	14	46.67%
Grade 4	5	16.67%
Histological grade of OSMF		
Grade 1	6	20%
Grade 2	15	50%
Grade 3	5	16.67%
Grade 4	4	13.3%

Among the study participants, fibrotic changes of the oral mucosa were most frequently observed in the bilateral buccal mucosa, affecting 12 (40%) patients. This was followed by involvement of the inner aspect of the upper and lower lips in 9 (30%), the bilateral buccal mucosa with the soft palate in 6 (20%), and the buccal mucosa along with the lips in 3 (10%) cases. Vesicular or ulcerative lesions were uncommon, with only one case observed on the soft palate.

The OSMF patients were clinically graded according to Mehrotra's clinical classification and histologically categorised using Khanna and Andrade's criteria. Clinically, the majority of patients 14 (46.6%) presented with Grade 3 OSMF, while histopathological assessment revealed that most patients 15 (50%) were classified as Grade 2 or lower.

**Figure 3:**
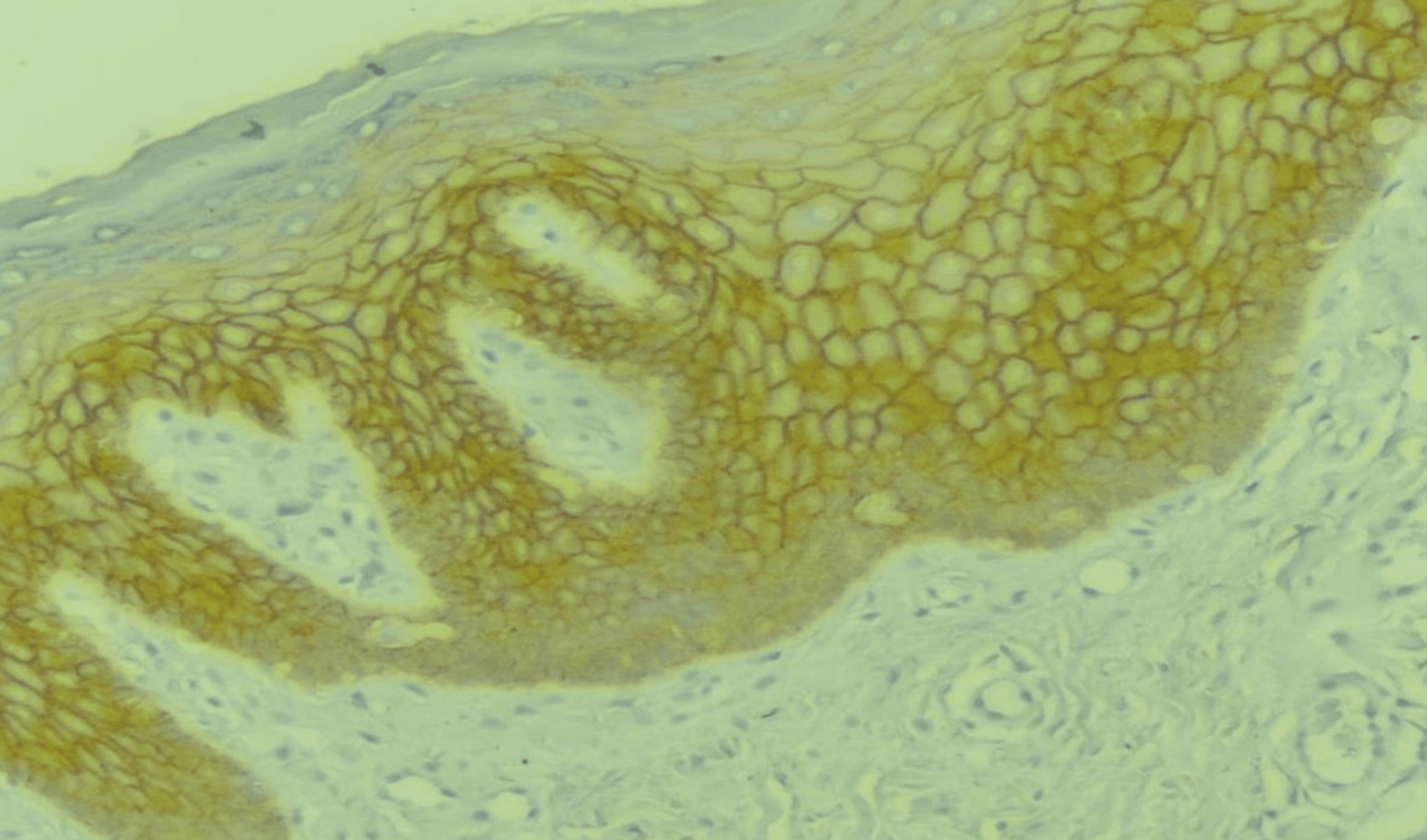
Immunohistochemical expression of E-cadherin in oral submucous fibrosis (DAB; 400x). DAB: diaminobenzidine.

**Figure 4:**
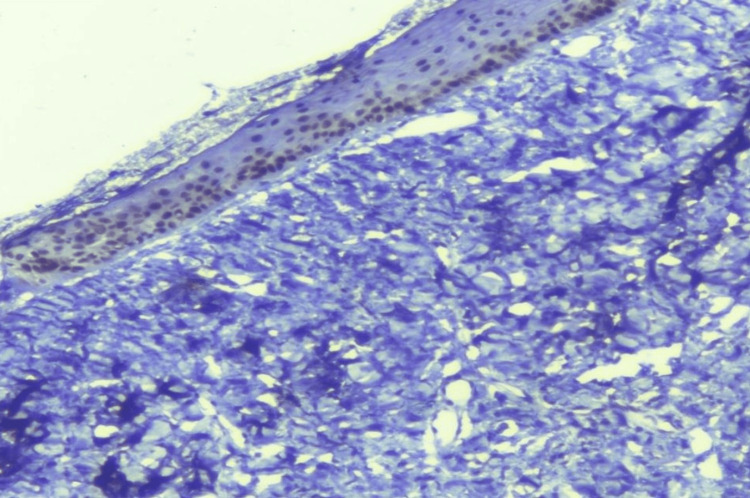
Basal and suprabasal expression of p63 in oral submucous fibrosis (DAB; 100x). DAB: diaminobenzidine.

Immunohistochemical analysis demonstrated distinct expression patterns of molecular markers across normal oral mucosa, oral submucous fibrosis (OSMF), and oral squamous cell carcinoma (OSCC) (Table 3). E-cadherin expression showed a sequential reduction from normal mucosa to OSCC, with strong membranous staining (4+) observed in all normal mucosa samples 5 (100%), compared to 14 (46.7%) in OSMF and complete absence in OSCC. Lower expression levels (1+ to 2+) were more frequently seen in OSCC 2 (40%), and this difference among groups was statistically significant (p = 0.017). In contrast, p63 expression showed a marked and statistically significant increase from normal mucosa to OSMF and OSCC (p < 0.001). Within the OSMF group, moderate to strong (3+) nuclear staining in the basal and suprabasal layers was observed in 26 (86.6%) patients, while intense nuclear expression (4+) was seen in 4 (80%) OSCC cases, indicating progressive epithelial proliferation across the disease spectrum (Figures 3, 4).

Similarly, SOX2 expression followed an upward trend from normal mucosa to OSMF and OSCC (p < 0.001). Strong nuclear staining (3+ and 4+) was confined mainly to OSCC, whereas OSMF showed moderate expression (2+ to 3+) with both basal and suprabasal distribution. Ki-67, a proliferation marker, also demonstrated a significant stepwise increase in expression (p < 0.001), with 6 (80%) of OSCC cases showing strong nuclear positivity compared to predominantly mild to moderate staining in OSMF and normal mucosa. Together, these findings indicate sequential molecular alterations from normal epithelium to premalignant and malignant states, with statistically significant differences across groups for all markers assessed.

**Table 3:** Comparison of immunohistochemical marker expression among normal oral mucosa, oral submucous fibrosis (OSMF), and oral squamous cell carcinoma (OSCC). The data are presented in the form of n (%). B: basal layer of epithelium, S: suprabasal layer of epithelium. *p-values less than 0.05 were considered statistically significant.

Characteristics	Level of expression	OSCC	Normal mucosa	OSMF	p-value
E-cadherin	1+	1 (20.00%)	0 (0.00%)	1 (3.33%)	0.017
	2+	1 (20.00%)	0 (0.00%)	3 (10.00%)	
	3+	2 (40.00%)	0 (0.00%)	12 (40.00%)	
	4+	0 (0.00%)	5 (100.00%)	14 (46.67%)	
	Neg	1 (20.00%)	0 (0.00%)	0 (0.00%)	
P63	1+(B,S)	0 (0.00%)	0 (0.00%)	1 (3.33%)	<0.001*
	1+(B)	0 (0.00%)	1 (20.00%)	0 (0.00%)	
	2+(B,S)	0 (0.00%)	3 (60.00%)	7 (23.33%)	
	3+	1 (20.00%)	0 (0.00%)	0 (0.00%)	
	3+(B,S)	0 (0.00%)	1 (20.00%)	19 (63.33%)	
	4+	4 (80.00%)	0 (0.00%)	0 (0.00%)	
	4+(B,S)	0 (0.00%)	0 (0.00%)	3 (10.00%)	
SOX2	1+(B)	0 (0.00%)	1 (20.00%)	6 (20.00%)	<0.001*
	2+	1 (20.00%)	0 (0.00%)	0 (0.00%)	
	2+(B,S)	0 (0.00%)	3 (60.00%)	6 (20.00%)	
	2+(B)	0 (0.00%)	0 (0.00%)	6 (20.00%)	
	3+	2 (40.00%)	0 (0.00%)	0 (0.00%)	
	3+(B,S)	0 (0.00%)	1 (20.00%)	10 (33.33%)	
	4+	2 (40.00%)	0 (0.00%)	0 (0.00%)	
	4+(B,S)	0 (0.00%)	0 (0.00%)	2 (6.67%)	
Ki-67	1+(B)	0 (0.00%)	3 (60.00%)	5 (16.67%)	<0.001*
	2+(B,S)	0 (0.00%)	1 (20.00%)	19 (63.33%)	
	2+(B)	0 (0.00%)	1 (20.00%)	1 (3.33%)	
	3+	4 (80.00%)	0 (0.00%)	0 (0.00%)	
	3+(B,S)	0 (0.00%)	0 (0.00%)	5 (16.67%)	
	4+	1 (20.00%)	0 (0.00%)	0 (0.00%)	

When correlated with the clinical grades of OSMF, however, no statistically significant association was observed for any of the markers studied (Table 4). E-cadherin expression remained relatively uniform across grades, with most cases showing moderate to strong (3+ to 4+) positivity (p = 0.8). Similarly, p63 and SOX2 showed variable but non-significant changes (p = 0.6 and p = 0.3, respectively), and Ki-67 expression remained comparable across all grades (p > 0.9). Collectively, all four biomarkers demonstrate significant alterations across the spectrum of normal mucosa to OSMF to OSCC; however, no significant differences in expression are noted within the spectrum of OSMF severity.

**Table 4:** Correlation of molecular marker expression with clinical grades of oral submucous fibrosis (OSMF). The data are presented in the form of n (%). B: basal layer of epithelium, S: suprabasal layer of epithelium. *p-values less than 0.05 were considered statistically significant.

Characteristic	Level of expression	Grade 1	Grade 2	Grade 3	Grade 4	p-value
E-cadherin	1+	1 (20.00%)	0 (0.00%)	0 (0.00%)	0 (0.00%)	0.8
	2+	0 (0.00%)	0 (0.00%)	3 (21.43%)	0 (0.00%)	
	3+	2 (40.00%)	3 (50.00%)	5 (35.71%)	2 (40.00%)	
	4+	2 (40.00%)	3 (50.00%)	6 (42.86%)	3 (60.00%)	
P63	1+(B,S)	0 (0.00%)	1 (16.67%)	0 (0.00%)	0 (0.00%)	0.6
	2+(B,S)	2 (40.00%)	0 (0.00%)	4 (28.57%)	1 (20.00%)	
	3+(B,S)	3 (60.00%)	5 (83.33%)	8 (57.14%)	3 (60.00%)	
	4+(B,S)	0 (0.00%)	0 (0.00%)	2 (14.29%)	1 (20.00%)	
SOX2	1+(B)	2 (40.00%)	1 (16.67%)	3 (21.43%)	0 (0.00%)	0.3
	2+(B,S)	0 (0.00%)	1 (16.67%)	4 (28.57%)	1 (20.00%)	
	2+(B)	0 (0.00%)	1 (16.67%)	2 (14.29%)	3 (60.00%)	
	3+(B,S)	3 (60.00%)	3 (50.00%)	4 (28.57%)	0 (0.00%)	
	4+(B,S)	0 (0.00%)	0 (0.00%)	1 (7.14%)	1 (20.00%)	
Ki-67	1+(B)	1 (20.00%)	1 (16.67%)	3 (21.43%)	0 (0.00%)	>0.9
	2+(B,S)	3 (60.00%)	4 (66.67%)	7 (50.00%)	5 (100.00%)	
	2+(B)	0 (0.00%)	0 (0.00%)	1 (7.14%)	0 (0.00%)	
	3+(B,S)	1 (20.00%)	1 (16.67%)	3 (21.43%)	0 (0.00%)	

Biochemical evaluation revealed significant differences in protein parameters but not in iron indices among the study groups (Table 5). Mean serum iron, total iron-binding capacity (TIBC), ferritin, and transferrin saturation values showed no statistically significant variation (p > 0.05). However, total serum protein levels declined sequentially from normal mucosa (7.96 ± 0.20 g/dL) to OSMF (7.51 ± 0.71 g/dL) and OSCC (6.89 ± 0.34 g/dL), with a significant difference among groups (p = 0.006). Albumin levels were also significantly reduced in OSCC compared to normal mucosa (p = 0.010). At the same time, the albumin-to-globulin ratio showed a significant alteration (p = 0.024), reflecting nutritional and metabolic imbalance associated with disease progression.

**Table 5:** Comparison of biochemical markers among normal mucosa, oral submucous fibrosis (OSMF), and oral squamous cell carcinoma (OSCC). Data are expressed as mean (SD). *p-values less than 0.05 were considered statistically significant. TIBC: total iron-binding capacity.

Characteristic	OSCC	Normal mucosa	OSMF	p-value
Serum iron	95.64 (15.77)	93.65 (22.67)	99.62 (41.69)	0.9
TIBC	399.01 (49.77)	385.98 (19.54)	401.65 (52.14)	0.6
Ferritin	61.11 (31.96)	55.27 (26.88)	91.11 (69.03)	0.4
Transferrin saturation	24.61 (13.29)	22.43 (7.21)	26.43 (11.88)	0.8
Total serum protein	6.89 (0.34)	7.96 (0.20)	7.51 (0.71)	0.006
Albumin	3.78 (0.25)	4.34 (0.37)	4.57 (0.48)	0.010*
Globulin	3.11 (0.32)	3.63 (0.35)	2.95 (0.70)	0.059
Albumin: Globulin ratio	1.22 (0.17)	1.21 (0.21)	1.70 (0.65)	0.024

Further analysis of biochemical parameters across OSMF grades revealed no statistically significant correlations (Table 6). Although minor fluctuations were noted in serum iron, ferritin, and total serum protein levels, none of the parameters varied significantly with clinical grading (p > 0.05). These results indicate that while biochemical changes occur during disease progression, they do not reflect the clinical severity of OSMF.

**Table 6:** Correlation of biochemical parameters with clinical grades of oral submucous fibrosis. Data are expressed as mean (SD). *p-values less than 0.05 were considered significant. TIBC: total iron-binding capacity.

Characteristic	Grade 1	Grade 2	Grade 3	Grade 4	p-value
Serum iron	109.62 (55.90)	74.11 (19.30)	113.71 (44.87)	80.80 (15.01)	0.066
TIBC	403.59 (33.51)	405.96 (14.60)	397.04 (71.89)	407.47 (37.07)	>0.9
Ferritin	108.02 (111.10)	53.43 (29.86)	109.06 (71.21)	69.12 (19.64)	0.2
Transferrin saturation	30.13 (10.45)	21.58 (11.86)	28.86 (13.94)	21.75 (2.45)	0.3
Total serum protein	7.42 (0.25)	7.68 (0.72)	7.61 (0.90)	7.15 (0.22)	0.12
Albumin	4.58 (0.28)	4.73 (0.22)	4.53 (0.63)	4.47 (0.41)	0.8
Globulin	2.85 (0.46)	2.95 (0.76)	3.07 (0.85)	2.68 (0.37)	0.3
Albumin: Globulin ratio	1.65 (0.39)	1.74 (0.65)	1.69 (0.83)	1.71 (0.36)	0.8

Ultrasonographic assessment of the masticatory and perioral muscles demonstrated a clear trend of muscle atrophy with advancing clinical grades of OSMF (Table 7). The mean thickness of the right masseter muscle decreased significantly from 11.00 ± 1.14 mm in Grade 1 to 8.54 ± 0.36 mm in Grade 4 (p = 0.001). Similar patterns were observed for the left masseter (p = 0.013), right and left temporalis (p = 0.024 and p = 0.004, respectively), and both sides of the orbicularis oris (p < 0.05). This sequential reduction in muscle thickness reflects the structural and functional involvement of deeper musculature in advanced stages of OSMF.

**Table 7:** Correlation between ultrasonographic muscle thickness and clinical grades of oral submucous fibrosis. Data are expressed as mean (SD). *p-values less than 0.05 were considered significant.

Characteristic	Grade 1	Grade 2	Grade 3	Grade 4	p-value
Thickness (right masseter)	11.00 (1.14)	10.78 (1.12)	9.34 (0.97)	8.54 (0.36)	0.001*
Thickness (left masseter)	10.08 (1.21)	9.95 (0.91)	9.35 (0.84)	8.26 (0.43)	0.013
Thickness (right temporalis)	7.10 (1.06)	7.10 (1.92)	5.43 (1.21)	4.82 (0.86)	0.024
Thickness (left temporalis)	7.00 (0.74)	7.58 (2.22)	5.60 (1.10)	4.50 (0.47)	0.004
Thickness (right orbicularis oris)	3.72 (0.26)	3.65 (0.52)	3.39 (0.46)	2.88 (0.37)	0.027
Thickness (left orbicularis oris)	3.50 (0.19)	3.38 (0.23)	3.59 (0.51)	2.78 (0.33)	0.017

When compared across normal mucosa, OSMF, and OSCC, ultrasonographic measurements revealed significant differences primarily in the temporalis muscle (Table 8). The right temporalis thickness was notably reduced in OSMF (5.94 ± 1.55 mm) compared to normal mucosa (6.88 ± 0.76 mm) (p = 0.047). Although the masseter and orbicularis oris muscles also demonstrated reduced thickness in OSMF and OSCC, these differences were not statistically significant (p > 0.05).

**Table 8:** Comparison of ultrasonographic muscle thickness among normal oral mucosa, oral submucous fibrosis, and oral squamous cell carcinoma. Data are expressed as mean (SD). *p-values less than 0.05 were considered significant. OSMF: oral submucous fibrosis; OSCC: oral squamous cell carcinoma; Rt: right; Lt: left.

Characteristic	N	OSCC	Normal mucosa	OSMF	p-value
Thickness (Rt masseter)	40	9.62 (0.50)	10.48 (0.45)	9.77 (1.29)	0.3
Thickness (Lt masseter)	40	9.82 (0.30)	10.40 (0.46)	9.41 (1.03)	0.10
Thickness (Rt temporalis)	40	7.36 (1.12)	6.88 (0.76)	5.94 (1.55)	0.047*
Thickness (Lt temporalis)	40	6.94 (0.83)	6.22 (0.64)	6.05 (1.63)	0.2
Thickness (Rt orbicularis oris)	38	3.13 (0.15)	4.12 (0.84)	3.41 (0.50)	0.079
Thickness (Lt orbicularis oris)	38	3.23 (0.25)	3.56 (0.30)	3.40 (0.48)	0.4

Needle electromyographic (EMG) analysis revealed sequential alterations in muscle activity across clinical grades of oral submucous fibrosis (OSMF) (Table 9). The mean motor unit potential duration in the right masseter decreased from 8.37 ± 2.90 ms in Grade 1 to 4.15 ± 1.80 ms in Grade 4 (p = 0.072), suggesting advancing myopathic changes. The left temporalis muscle showed a significant increase in potential duration with disease severity (p = 0.002), while left masseter amplitude also varied significantly (p = 0.003), indicating altered motor unit recruitment. Other muscles exhibited non-significant fluctuations, reflecting variable involvement of masticatory muscles with progressive fibrosis.

**Table 9:** Correlation of needle electromyography findings with clinical grades of oral submucous fibrosis. Data are expressed as mean (SD). *p-values less than 0.05 were considered significant. Rt: right; Lt: left.

Characteristic	Grade 1	Grade 2	Grade 3	Grade 4	p-value
Needle duration (Rt masseter mean)	8.37 (2.90)	5.08 (1.08)	4.33 (0.89)	4.15 (1.80)	0.072
Needle amplitude (Rt masseter mean)	442.50 (263.40)	792.83 (546.70)	610.64 (300.44)	540.99 (310.09)	0.8
Needle duration (Lt masseter mean)	5.02 (0.19)	5.80 (1.56)	4.99 (1.35)	6.40 (0.58)	0.062
Needle amplitude (Lt masseter mean)	394.80 (206.06)	730.75 (357.69)	567.61 (274.34)	1,251.20 (313.44)	0.003*
Needle duration (Rt temporalis mean)	7.96 (1.63)	5.88 (0.87)	6.43 (1.98)	5.44 (1.51)	0.11
Needle amplitude (Rt temporalis mean)	777.40 (144.78)	551.58 (333.86)	666.64 (447.39)	362.30 (130.67)	0.094
Needle duration (Lt temporalis mean)	4.56 (0.12)	5.07 (0.72)	6.08 (1.49)	9.62 (3.05)	0.002*
Needle amplitude (Lt temporalis mean)	456.50 (294.26)	457.42 (85.67)	722.61 (428.87)	1,018.20 (891.65)	0.4
Needle duration (Rt orbicularis mean)	5.25 (0.87)	4.89 (2.27)	5.85 (4.07)	4.18 (3.35)	0.6
Needle amplitude (Rt orbicularis mean)	395.20 (154.21)	532.58 (468.93)	616.25 (265.48)	885.10 (630.68)	0.4
Needle duration (Lt orbicularis mean)	4.74 (0.58)	4.46 (0.94)	5.04(1.14)	5.91 (2.59)	0.7
Needle amplitude (Lt orbicularis mean)	400.60 (285.24)	569.83 (417.59)	522.79 (286.93)	478.70 (282.41)	0.7

When compared with normal mucosa, OSMF patients showed significantly reduced left masseter amplitude (685.37 ± 389.98 µV vs. 1083.90 ± 319.14 µV; p = 0.043), indicating compromised motor unit activity (Table 10). The right orbicularis oris muscle demonstrated a longer mean potential duration in OSMF (5.28 ± 3.22 ms) than controls (2.80 ± 1.01 ms; p = 0.062), though not statistically significant. Overall, EMG findings indicate early electrophysiological evidence of muscle dysfunction in OSMF, characterised by reduced amplitude and altered duration patterns consistent with progressive fibrotic and myopathic changes.

**Table 10:** Comparison of needle electromyography parameters among normal oral mucosa and oral submucous fibrosis. Data are expressed as mean (SD). *p-values less than 0.05 were considered significant. OSMF: oral submucous fibrosis.

Characteristic	Normal mucosa	OSMF	p-value
Needle duration (Rt masseter mean)	4.76 (1.08)	5.12 (2.11)	>0.9
Needle amplitude (Rt masseter mean)	659.90 (176.77)	607.45 (356.92)	0.4
Needle duration (Lt masseter mean)	4.48 (1.14)	5.39 (1.26)	0.2
Needle amplitude (Lt masseter mean)	1,083.90 (319.14)	685.37 (389.98)	0.043*
Needle duration (Rt temporalis mean)	5.54 (1.10)	6.41 (1.79)	0.3
Needle amplitude (Rt temporalis mean)	863.30 (421.40)	611.37 (363.12)	0.2
Needle duration (Lt temporalis mean)	5.69 (0.95)	6.21 (2.26)	0.9
Needle amplitude (Lt temporalis mean)	777.90 (382.63)	674.48 (494.72)	0.3
Needle duration (Rt orbicularis mean)	2.80 (1.01)	5.28 (3.22)	0.062
Needle amplitude (Rt orbicularis mean)	484.30 (127.36)	607.48 (387.18)	0.8
Needle duration (Lt orbicularis mean)	4.24 (1.04)	7.5 (1.2)	0.2
Needle amplitude (Lt orbicularis mean)	653.40 (169.75)	504.48 (303.67)	0.14

## Discussion

The present study provides an integrated understanding of the molecular, biochemical, and functional alterations involved in the pathogenesis and progression of oral submucous fibrosis (OSMF). The malignant transformation potential of OSMF has been linked to multiple factors, including the duration and frequency of areca nut use, age of onset, gender, and variations in diagnostic criteria [13-15]. Despite several proposed clinical and histopathological classifications, the biological mechanisms underlying malignant transformation remain incompletely understood. Recent studies have explored molecular markers, such as p63, Ki-67, SOX2, and E-cadherin, as early indicators of epithelial instability and carcinogenic progression in OSMF [16,17].

In the present study, E-cadherin expression sequentially decreased from normal oral mucosa to OSMF and oral squamous cell carcinoma. This pattern aligns with the literature, which shows that membranous E-cadherin expression progressively decreases with increasing epithelial dysplasia and malignant transformation [16,18]. Although no statistically significant correlation was noted between E-cadherin expression and clinical or histologic grades of OSMF, its overall reduction across disease stages suggests its potential role as an indicator of malignant transformation rather than a marker of disease severity.

The expression of p63 gradually increased from normal mucosa to OSMF to OSCC, with predominantly basal and supra-basal staining patterns in OSMF. These findings are in accordance with reports by Suwasini et al. and Sinha et al., who demonstrated similar trends in p63 expression [19,20]. However, no statistically significant correlation was observed between p63 expression and disease grades, which may be due to moderate expression even in early stages. This suggests that p63 upregulation may indicate proliferative activity and early epithelial dysregulation, supporting its utility as a biomarker for malignant potential rather than for grading OSMF severity.

Similarly, Ki-67, a nuclear proliferation marker, showed an increased expression from normal mucosa to OSMF to OSCC. This trend aligns with earlier observations by Iqbal et al. and Humayun et al., who also noted similar expression patterns of Ki-67 in oral premalignant lesions [21,22]. Although our results did not reveal a significant association between Ki-67 expression and OSMF grades, the overall trend suggests that the oral mucosal epithelium in OSMF exhibits a higher proliferative potential compared to normal mucosa, indicating early neoplastic changes. Even in the absence of histological dysplasia, elevated Ki-67 and p63 expression suggest that molecular alterations precede observable epithelial dysplasia in OSMF.

SOX2 is a stem cell transcription factor essential for epithelial renewal. It was upregulated in OSMF compared to normal mucosa, with the highest expression seen in OSCC. This observation is consistent with the findings of Chandran et al., who reported elevated SOX2 expression in OSMF with dysplasia [23]. The gradual increase in SOX2 expression across the normal-OSMF-OSCC spectrum highlights its potential role in maintaining stemness and initiating early carcinogenic pathways, even in non-dysplastic OSMF tissue.

Biochemical evaluation revealed no significant correlation between serum iron or total protein levels and either clinical or histologic grades of OSMF. However, when compared across diagnostic groups, total serum protein levels were significantly reduced from normal mucosa to OSMF to OSCC (p = 0.006), consistent with previous studies suggesting altered collagen metabolism and systemic nutritional imbalance in OSMF patients [24]. Although serum iron, ferritin, and transferrin saturation showed no significant variation, these findings align with previous reports by Bhardwaj et al. and Thakur et al., who also observed variable trends among patients with OSMF [25,26].

Ultrasonographic evaluation of masticator muscles revealed a significant reduction in the thickness of the masseter, temporalis, and orbicularis oris muscles with increasing clinical grade. A similar study by Kant et al. also noted an early involvement of the masseter muscle in patients with OSMF [8]. This reduction likely reflects disuse atrophy due to restricted mouth opening and progressive fibrosis. Electromyographic (EMG) analysis demonstrated a general reduction in motor unit action potential duration, particularly in the right masseter, indicating myopathic changes associated with fibrotic infiltration. Interestingly, the left temporalis muscle showed a significant increase in duration with advancing grades, likely reflecting compensatory recruitment in response to restricted jaw mobility. These findings suggest that both mechanical and electrophysiological alterations contribute to functional limitation, as evidenced by muscle thinning and altered motor unit activity.

Overall, our findings demonstrate that p63, Ki-67, SOX2, and E-cadherin exhibit sequential molecular alterations from normal mucosa through OSMF to OSCC, reflecting their potential roles in malignant transformation. However, their expression did not correlate with clinical or histological severity, indicating that these markers are more useful as potential indicators of carcinogenic risk than as indicators of disease stage. Similarly, the biochemical and functional findings emphasise the multifactorial nature of OSMF pathogenesis involving both epithelial and muscular components. Future studies with larger cohorts and longitudinal follow-up are warranted to validate these biomarkers as prognostic tools for early detection and risk stratification in OSMF.

Limitations

This study has several limitations that must be considered when interpreting the findings. The sample size, although sufficient for exploratory analysis, was relatively small, particularly within the control and OSCC groups, limiting the statistical power to detect finer subgroup differences. The cross-sectional design prevents assessment of disease progression over time or the ability to link biomarker alterations with eventual malignant transformation. Recruitment from a single tertiary care centre may introduce selection bias, and variability in patient habits, such as frequency, duration, and type of areca nut or tobacco use, could act as uncontrolled confounding factors affecting clinical, biochemical, and molecular outcomes. Additionally, interobserver variability in ultrasonographic and electromyographic measurements cannot be entirely excluded despite standardised protocols. Despite these limitations, the study provides valuable multidimensional insights into the behaviour of OSMF and establishes groundwork for future longitudinal research.

## Conclusions

This study provides a multidimensional perspective on oral submucous fibrosis by integrating immunohistochemical, biochemical, ultrasonographic, and electromyographic assessments. Sequential epithelial and muscular alterations were observed alongside molecular changes, including decreased E-cadherin and increased p63, Ki-67, and SOX2 expression from normal mucosa to OSMF to OSCC, suggesting potential early events in malignant transformation. Serum iron and total protein levels showed no significant association with clinical grades, while ultrasound and EMG indicated muscle atrophy and reduced activity contributing to trismus. Although these findings enhance understanding of OSMF pathophysiology, they are observational and do not establish causality or definitive predictive value. Larger, longitudinal studies are needed to validate these associations and determine their clinical relevance.
